# Challenges and Prospects in Vision and Language Research

**DOI:** 10.3389/frai.2019.00028

**Published:** 2019-12-13

**Authors:** Kushal Kafle, Robik Shrestha, Christopher Kanan

**Affiliations:** ^1^Center for Imaging Science, Rochester Institute of Technology, Rochester, NY, United States; ^2^Paige, New York, NY, United States; ^3^Cornell Tech, New York, NY, United States

**Keywords:** computer vision, natural language understanding, visual question answering, captioning, dataset bias, visual Turing test

## Abstract

Language grounded image understanding tasks have often been proposed as a method for evaluating progress in artificial intelligence. Ideally, these tasks should test a plethora of capabilities that integrate computer vision, reasoning, and natural language understanding. However, the datasets and evaluation procedures used in these tasks are replete with flaws which allows the vision and language (V&L) algorithms to achieve a good performance without a robust understanding of vision and language. We argue for this position based on several recent studies in V&L literature and our own observations of dataset bias, robustness, and spurious correlations. Finally, we propose that several of these challenges can be mitigated by creation of carefully designed benchmarks.

## 1. Introduction

Advancements in deep learning and the availability of large-scale datasets have resulted in great progress in computer vision and natural language processing (NLP). Deep convolutional neural networks (CNNs) have enabled unprecedented improvements in classical computer vision tasks, e.g., image classification (Russakovsky et al., 2015) and object detection (Lin et al., 2014). Similarly, various deep learning based approaches have enabled enormous advances in classical NLP tasks, e.g., named entity recognition (Yadav and Bethard, 2018), sentiment analysis (Zhang et al., 2018b), question-answering (Saeidi et al., 2018; Reddy et al., 2019), and dialog systems (Chen et al., 2017). Building upon these advances, there is a push to attack new problems that enable concept comprehension and reasoning capabilities to be studied at the intersection of vision and language (V&L) understanding. There are numerous applications for V&L systems, including enabling the visually impaired to interact with visual content using language, human-computer interaction, and visual search. Human-robot collaboration would be greatly enhanced by giving robots understanding of human language to better understand the visual world.

However, the primary objective of many scientists working on V&L problems is to have them serve as stepping stones toward a visual Turing test (Geman et al., 2015), a benchmark for progress in artificial intelligence (AI). To pass the visual Turing test, a V&L algorithm must demonstrate a robust understanding of natural language and an ability to visually ground the linguistic concepts in the form of objects, their attributes, and their relationships.

Integrating vision and language provides a test-bed for assessing both natural language understanding and goal-directed visual understanding. V&L tasks can demand many disparate computer vision and NLP skills to be used simultaneously. For example, the same system may be required to simultaneously engage in entity extraction, entailment and co-reference resolution, visual and linguistic reasoning, object recognition, attribute detection, and much more. Most V&L benchmarks capture only a fraction of the requirements of a rigorous Turing test; however, we argue that a rigorous evaluation should test each capability required for visual and linguistic understanding *independently*, which will help in assessing if an algorithm is right for the right reasons. If it is possible to do well on a benchmark by ignoring visual and/or linguistic inputs, or by merely guessing based on spurious correlations, then it will not satisfy these requisites for a good test.

Many V&L tasks have been proposed, including image and video captioning (Mao et al., 2015; Yu et al., 2016), visual question answering (VQA) (Antol et al., 2015; Zhang et al., 2016; Agrawal et al., 2017, 2018; Kafle and Kanan, 2017a,b), referring expression recognition (RER) (Kazemzadeh et al., 2014), image retrieval (Mezaris et al., 2003; Johnson et al., 2015), activity recognition (Yatskar et al., 2016; Zhao et al., 2017a), and language-guided image generation (Reed et al., 2016; Zhang et al., 2017). A wide variety of algorithms have been proposed for each of these tasks, producing increasingly better results across datasets. However, several studies have called into question the *true* capability of these systems and the efficacy of current assessment methods (Kafle and Kanan, 2017a; Cirik et al., 2018; Madhyastha et al., 2018). Systems are heavily influenced by dataset bias and lack robustness to uncommon visual configurations (Agrawal et al., 2017; Kafle and Kanan, 2017a; Madhyastha et al., 2018), but these are often not measured and call into question the value of these benchmarks. These issues also impact system assessment and deployment. Systems can amplify spurious correlations between gender and potentially unrelated variables in V&L problems (Zhao et al., 2017a; Hendricks et al., 2018), resulting in the possibility of severe negative real-world impact.

In this article, we outline the current state of V&L research. We identify the challenges in developing good algorithms, datasets, and evaluation metrics. We discuss issues unique to individual tasks as well as identify common shortcomings shared across V&L benchmarks. Finally, we provide our perspective on potential future directions for V&L research. In particular, we argue that both content and evaluation procedure of future V&L benchmarks should be carefully designed to mitigate dataset bias and superficial correlations. To this end, we propose a few concrete steps for the design of future V&L tasks that will serve as robust benchmarks for measuring progress in natural language understanding, computer vision, and the intersection of the two.

## 2. A Brief Survey of V&L Research

**Figure 1:**
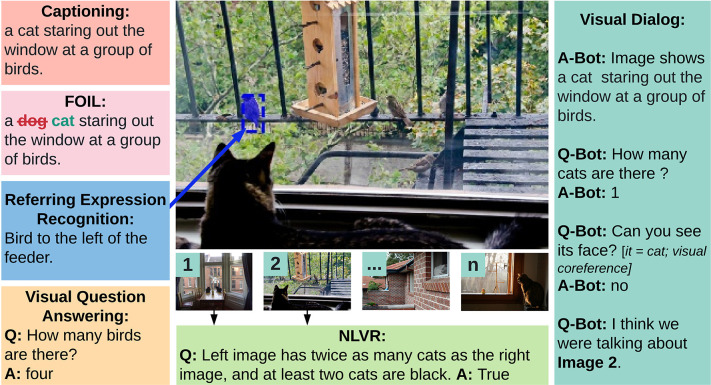
Common tasks in vision and language research.

Multiple V&L tasks have been proposed for developing and evaluating AI systems. We briefly describe the most prominent V&L tasks and discuss baseline and state-of-the-art algorithms. Some of these tasks are shown in Figure 1.

### 2.1. Tasks in V&L Research

Bidirectional sentence-to-image and image-to-sentence retrieval problems are among the earliest V&L tasks (Mezaris et al., 2003). Early works dealt with simpler keyword-based image retrieval (Mezaris et al., 2003), with later approaches using deep learning and graph-based representations (Johnson et al., 2015). Visual semantic role labeling requires recognizing activities and semantic context in images (Yatskar et al., 2016; Zhao et al., 2017a). Image captioning, the task of generating descriptions for visual content, involves both visual and language understanding. It requires describing the gist of the *interesting* content in a scene (Lin et al., 2014; Donahue et al., 2015), while also capturing specific image regions (Johnson et al., 2016). Video captioning adds the additional complexity of understanding temporal relations (Yu et al., 2016). Unfortunately, it is difficult to evaluate the quality and relevance of generated captions without involving humans (Elliott and Keller, 2014). Automatic evaluation metrics (Papineni et al., 2002; Lin, 2004) are incapable of assigning due merit to the large range of valid and relevant descriptions for visual content and are poorly correlated with human judgment, often ranking machine-generated captions as being better than human captions (Bernardi et al., 2016; Kilickaya et al., 2017).

VQA involves answering questions about visual content. Compared to captioning, it is better suited for automatic evaluation as the output can be directly compared against ground truth answers as long as the answers are one or perhaps two words long (Antol et al., 2015; Kumar et al., 2016; Goyal et al., 2017). VQA was proposed as a form of visual Turing test, since answering arbitrary questions could demand many different skills to facilitate scene understanding. While many believed VQA would be extremely challenging, results on the first natural image datasets quickly rivaled humans, which was in large part due to question-answer distribution bias being ignored in evaluation (Agrawal et al., 2016, 2017, 2018; Zhang et al., 2016; Kafle and Kanan, 2017a). Results were good for common questions, but systems were fragile and were incapable of handling rare questions or novel scenarios. Later datasets attempted to better assess generalization. The Task Directed Image Understanding Challenge (TDIUC) tests generalization to multiple question-types (Kafle and Kanan, 2017a), Compositional VQA (C-VQA) evaluates the ability to handle novel concept compositions (Agrawal et al., 2017), and VQA under Changing Priors (VQA-CP) tests generalization to different answer distributions (Agrawal et al., 2018). It is harder to excel on these datasets by just exploiting biases. However, the vast majority of the questions in these datasets do not require complex compositional reasoning. The CLEVR dataset attempts to address this by generating synthetic questions demanding complex chains of reasoning about synthetic scenes consisting of simple geometric shapes (Johnson et al., 2017a). Similar to CLEVR, the GQA dataset measures compositional reasoning in natural images by asking long and complex questions in visual scenes involving real-world complexities (Hudson and Manning, 2019). Video Question Answering has the additional requirement of understanding temporal dynamics (Zhao et al., 2017b; Zhu et al., 2017). We refer readers to survey articles for extensive reviews on VQA (Kafle and Kanan, 2017b) and image captioning (Bernardi et al., 2016).

With VQA, models do not have to provide visual evidence for their outputs. In contrast, RER requires models to provide evidence by either selecting among a list of possible image regions or generating bounding boxes that correspond to input phrases (Kazemzadeh et al., 2014; Rohrbach et al., 2016; Plummer et al., 2017). Since the output of an RER query is *always* a single box, it is often quite easy to *guess* the correct box. To counter this, Acharya et al. (2019) proposed visual query detection (VQD), a form of goal-directed object detection, where the query can have 0–15 valid boxes making the task more difficult and more applicable to real-world applications. FOIL takes a different approach and requires a system to differentiate invalid image descriptions from valid ones (Shekhar et al., 2017). Natural Language Visual Reasoning (NLVR) requires verifying if image descriptions are true (Suhr et al., 2017, 2018).

Unlike the aforementioned tasks, EmbodiedQA requires the agent to explore its environment to answer questions (Das et al., 2018). The agent must actively perceive and reason about its visual environment to determine its next actions. In visual dialog, an algorithm must hold a conversation about an image (Das et al., 2017a,b). In contrast to VQA, visual dialog requires understanding the conversation history, which may contain visual co-references that a system must resolve correctly. The idea of conversational visual reasoning has also been explored in Co-Draw (Kim et al., 2019), a task where a *teller* describes visual scenes and a *drawer* draws them without looking at the original scenes.

Of course, it is impossible to create an agent that knows everything about the visual world. Agents are bound to encounter novel situations, and handling these situations requires them to be aware of their own limitations. Visual curiosity addresses this by creating agents that pose questions to knowledgeable entities, e.g., humans or databases, and then they incorporate the new information for future use (Misra et al., 2018; Yang et al., 2018; Zhang et al., 2018a).

### 2.2. V&L Algorithms

In general, V&L algorithms have three sub-systems: (1) visual processing, (2) language processing, and (3) multi-modal integration.

For visual processing, almost all algorithms use CNN features. Typically, ImageNet pre-trained CNNs are used for natural scene datasets and shallow CNNs are used for synthetic scene datasets (Santoro et al., 2017). Until 2017, most algorithms for natural scenes used CNN features directly; however, more recent algorithms have switched to using CNN region proposal features (Anderson et al., 2018). Another recent trend is the use of graph-based representations for image retrieval (Johnson et al., 2015), image generation (Johnson et al., 2018), VQA (Yi et al., 2018), and semantic knowledge incorporation (Yi et al., 2018), due to their intuitiveness and suitability for symbolic reasoning.

For language representation, most V&L systems process words using recurrent neural networks (RNNs). For tasks that take queries as input, word tokens fed to the RNN are commonly learned as vector embeddings in an end-to-end manner with the network being trained on a downstream-task (Agrawal et al., 2018; Kim et al., 2018; Zhang et al., 2018a). Recent V&L systems leverage distributed representations of words trained on large corpora of natural language text. Common choices include word2vec (Mikolov and Dean, 2013), GloVe (Pennington et al., 2014), and fasttext (Singh et al., 2019). A few approaches have incorporated explicit syntax and semantic information from language, such as part-of-speech based semantic parsing (Agrawal et al., 2018) and dependency trees (Cao et al., 2018); however, distributed vector representations remain the dominant language representation for most recent systems.

A variety of approaches have been explored for fusing the outputs of the vision and language processing systems. Fusion mechanisms range from simple techniques, such as concatenation and Hadamard products (Antol et al., 2015; Kafle and Kanan, 2016), to more intricate methods, e.g., bilinear fusion (Fukui et al., 2016), which are argued to better capture interactions between visual and linguistic representations. Attention mechanisms that enable extraction of query-relevant information have also been heavily explored (Yang et al., 2016; Anderson et al., 2018; Kim et al., 2018; Yu et al., 2018). Attention mechanisms learn to assign higher *importance* to relevant information using both top-down and bottom-up pathways (Anderson et al., 2018).

Some V&L tasks require compositional reasoning mechanisms. Typically, these mechanisms enable multiple explicit processing steps for answering complex queries, e.g, recognizing visual objects, filtering query-relevant visual regions, and describing visual entities. Modular networks are one of the best known compositional reasoning mechanisms (Andreas et al., 2016; Hu et al., 2017; Yu et al., 2018). Compositional reasoning can also be achieved by capturing pairwise interactions between V&L representations (Santoro et al., 2017) and by recurrently extracting and consolidating information from the input (Hudson and Manning, 2018). These approaches directly learn reasoning from data by utilizing structural biases provided by the model definition.

While these algorithms show impressive new capabilities, their development and evaluation has been split into two distinct camps: the first camp focuses on monolithic architectures that often excel at natural image V&L tasks (Kim et al., 2016; Yang et al., 2016), whereas the second camp focuses on compositional architectures, that excel at synthetically generated scenes testing for compositional reasoning (Santoro et al., 2017; Hudson and Manning, 2018). Algorithms developed for one camp are often not evaluated on the datasets from other camp, which makes it difficult to gauge the true capabilities of V&L algorithms. Shrestha et al. (2019) showed that most of the algorithms developed for natural image VQA do not perform well on synthetic compositional datasets and vice-versa. The authors further propose a simple architecture that compares favorably against state-of-the-art algorithms from both camps, indicating that specialized mechanisms such as: attention, modular reasoning and fusion mechanisms, used in more intricate methods may been over-engineered to perform well on selected datasets.

## 3. Shortcomings of V&L Research

Progress in V&L research appears to be swift. For several V&L benchmarks, algorithms now rival human performance (Bernardi et al., 2016; Johnson et al., 2017b). However, these results are misleading because they ensue from the shortcomings in benchmarks rather than an algorithm's capability of true V&L understanding. In this section, we describe several such shortcomings.

### 3.1. Dataset Bias

Dataset bias is a serious challenge faced by both computer vision (Torralba and Efros, 2011; Tommasi et al., 2017) and NLP (Bolukbasi et al., 2016; Zhao et al., 2017a) systems. Because V&L systems operate at the intersection of the two, unwanted and unchecked biases are very prevalent in V&L tasks too. Since the data used for training and testing a model are often collected homogeneously (Lin et al., 2014; Antol et al., 2015; Goyal et al., 2017), they share common patterns and regularities. Hence, it is possible for an algorithm to get good results by memorizing those patterns, undermining our efforts to evaluate the understanding of vision and language. The biases in datasets can stem from several sources, can be hard to track, and can result in severely misleading model evaluation. Two of the most common forms of bias stem from bias in crowd-sourced annotators and naturally occurring regularities. Finally, “photographer's bias” is also prevalent in V&L benchmarks, because images found on the web share similarities in posture and composition due to humans having preferences for specific views (Azulay and Weiss, 2018). Since the same biases and patterns are also mirrored in the test dataset, algorithms can simply memorize these superficial patterns (If the question has the pattern “Is there an OBJECT in the picture?,” then answer “yes”) instead of learning to actually solve the intended task (answer “yes” only if the OBJECT is actually present). If this bias is not compensated for during evaluation, benchmarks may only test a very narrow subset of capabilities. This can enable algorithms to perform well for the wrong reasons and algorithms can end up catastrophically failing in uncommon scenarios (Agrawal et al., 2018; Alcorn et al., 2019).

**Figure 2:**
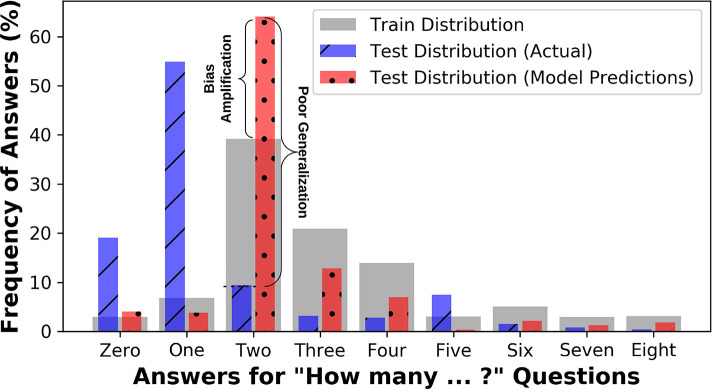
Answer distribution for questions starting with the phrase “How many” in the train and test splits of VQA-CP dataset (Agrawal et al., 2018), alongside the test-set predictions from a state-of-the-art VQA model, BAN (Kim et al., 2018). In VQA-CP, the distribution of test set is intentionally made different from the training set to assess if the algorithms can perform well under changing priors. Algorithms not only fail to perform well under changing priors, but they also demonstrate bias-amplification, i.e., the predictions show increased bias toward answers that are more common in the training set than the actual level of bias.

Several studies demonstrate the issue of bias in V&L tasks. For example, blind VQA models that “guess” the answers without looking at images show relatively high accuracy (Kafle and Kanan, 2016). In captioning, simple nearest neighbor-based approaches yield surprisingly good results (Devlin et al., 2015). Dataset bias occurs in other V&L tasks as well (Shekhar et al., 2017; Zhao et al., 2017a; Cirik et al., 2018; Zellers et al., 2018). Recent studies (Zhao et al., 2017a) have shown that algorithms not only *mirror* the dataset bias in their predictions, but in fact *amplify* the effects of bias (see Figure 2).

Numerous studies have sought to quantify and mitigate the effects of answer distribution bias on an algorithm's performance. As a straightforward solution, Zhang et al. (2016) and Kafle and Kanan (2017a) proposed balanced training sets with a uniform distribution over possible answers. This is somewhat effective for simple binary questions and synthetically generated visual scenes, but it does not address the imbalance in the kinds of questions present in the datasets. Re-balancing all kinds of query types is infeasible for large-scale natural image datasets. Furthermore, it may be counterproductive to forgo information contained in natural distributions in the visual and linguistic content, and focus should instead be on rigorous evaluation that compensates for bias or demonstrates bias robustness (Agrawal et al., 2018). We discuss this further in the next section.

### 3.2. Evaluation Metrics

Proper evaluation of V&L algorithms is difficult. In computer vision, challenges in evaluation can primarily be attributed to class imbalance and dataset bias (Godil et al., 2014; Buda et al., 2018). Evaluation of NLP algorithms often poses greater challenges since the notion of *goodness* is ill-defined for natural language. These challenges, especially in the automatic translation and natural language generation tasks (Novikova et al., 2017; Shimanaka et al., 2018), have been thoroughly documented in the NLP community. Unsurprisingly, these issues also translate to V&L tasks, and are often further exacerbated by the added requirement of V&L integration. In V&L tasks, language can be used to express similar visual semantic content in different ways, which makes automatic evaluation of models that emit words and sentences particularly challenging. For example, the captions “A man is walking next to a tree” and “A guy is taking a stroll by the tree” are nearly identical in meaning, but it can be hard for automatic systems to infer that fact. Several evaluation metrics have been proposed for captioning, including simple n-gram matching systems [e.g., BLEU (Papineni et al., 2002), CIDEr (Vedantam et al., 2015), and ROUGE (Lin, 2004)] and human consensus-based measures (Vedantam et al., 2015). Most of these metrics have limitations (Bernardi et al., 2016; Kilickaya et al., 2017), with n-gram based metrics suffering immensely for sentences that are phrased differently but have identical meaning or use synonyms (Kilickaya et al., 2017). Alarmingly, evaluation metrics often rank machine-generated captions as being better than human captions but fail when human subjectivity is taken into account (Bernardi et al., 2016; Kilickaya et al., 2017). Even humans find it hard to agree on what a “good” caption entails (Vedantam et al., 2015). Automatic evaluation of captioning is further complicated because it is not clear what is expected from the captioning system. A given image can have many valid captions ranging from descriptions of specific objects in an image, to an overall description of the entire image. However, due to natural regularities and photographer bias, generic captions can apply to a large number of images, thereby gaining high evaluation scores without demonstrating visual understanding (Devlin et al., 2015).

Evaluation issues are lessened in VQA and RER where the output is better defined; however, it is not completely resolved. If performance for VQA is measured using exact answer matches, then even small variations will be harshly punished, e.g., if a model predicts “bird” instead of “eagle,” then the algorithm is punished as harshly as if it were to predict “table.” Several solutions have been proposed, but they have their own limitations, e.g., Wu-Palmer Similarity (WUPS), a word similarity metric, cannot be used with sentences and phrases. Alternately, consensus based metrics have been explored (Antol et al., 2015; Malinowski et al., 2015), where multiple annotations are collected for each input, with the intention of capturing common variations of the ground truth answer. However, this paradigm can make many questions *unanswerable* due to low human consensus (Kafle and Kanan, 2016, 2017a). Multiple-choice evaluation has been proposed by several benchmarks (Antol et al., 2015; Goyal et al., 2017). While this simplifies evaluation, it takes away a lot of the open-world difficulty from the task and can lead to inflated performance via smart guessing (Jabri et al., 2016).

Dataset biases introduce further complications for evaluation metrics. Inadequate metrics can conflate the issues of bias when the statistical distributions of the training and test sets are not taken into account, artificially inflating performance. Metrics normalized to account for the distribution of training data (Kafle and Kanan, 2017a) and diagnostic datasets that artificially perturb the distribution of train and test data (Agrawal et al., 2018) have been proposed to remedy this. Furthermore, open-ended V&L language tasks can *potentially* test a variety of skills, ranging from relatively easy sub-tasks (detection of large, well-defined objects), to fairly difficult sub-tasks (fine-grained attribute detection, spatial and compositional reasoning, counting, etc.). However, these tasks are not evenly distributed. Placing all skill types on the same footing can inflate system scores and hide how fragile these systems are. Dividing the dataset into underlying tasks can help (Kafle and Kanan, 2017a), but the best way to make such a division is not clearly defined.

### 3.3. Are V&L Systems “Horses?”

Sturm defines a “horse” as **“a system that appears as if it is solving a particular problem when it actually is not”** (Sturm, 2016). Of course, the “horse” here refers to the infamous horse named Clever Hans, thought to be capable of arithmetic and abstract thought but was in reality exploiting the micro-signals provided by its handler and audience. Similar issues are prevalent in both computer vision and NLP, where it is possible for models to arrive at a correct answer by simply exploiting spurious statistical *cues* rather than through robust understanding of the underlying problem. This results in algorithms that achieve higher accuracy but are brittle when subjected to *stress-tests*. For example, in computer vision, CNNs trained on the Imagenet are shown to be biased toward textures rather than the shape resulting in poor generalization to distortions and sub-optimal object detection performance (Geirhos et al., 2019). In NLP, these issues are even more prevalent. Sharma et al. (2019) shows that it is possible to *guess* the correct answer in a conversational question-answering task by exploiting cues in the prior conversation for up-to 84% of the time. Similarly, in natural language inference (NLI), where the task is to determine whether a hypothesis is *neutral*, an *entailment*, or a *contradiction* to the given premise, a hypothesis-only baseline (which has not seen the premise) significantly outperforms majority-class baseline (Poliak et al., 2018). This shows that exploiting statistical *cues* contributes to inflated performance. Niven and Kao (2019) shows similar effects of spurious correlations in argument reasoning comprehension. As V&L research inherits from these research, similar issues are highly prevalent in V&L research. In this section, we review several of these issues and highlight existing studies that scrutinize the true capabilities of existing V&L systems to assess whether they are “horses.”

#### 3.3.1. Superficial Correlations and True vs. Apparent Difficulty

**Figure 3:**
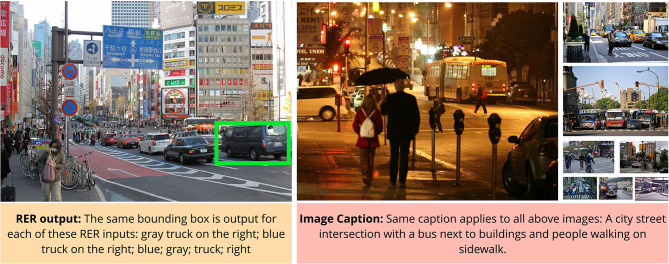
The apparent vs. true complexity of V&L tasks. In RER **(left)**, omitting a large amount of text has no effect on the output of the system (Yu et al., 2018). Similarly, a seemingly detailed caption **(right)** can apply to a large number of images from the dataset making it easy to “guess” based on shallow correlations. While it appears as though the captioning system can identify objects (“bus,” “building,” “people”), spatial relationships (“next to,” “on”), and activities (“walking”). However, it is entirely possible for the captioning system to have ‘guessed’ the caption by detection of one of the objects in the caption, e.g., a “bus” or even *a common latent* object such as “traffic light”.

Due to superficial correlations, the difficulty of V&L datasets may be much lower than the true difficulty of comprehensively solving the task (see Figure 3). We outline some of the key studies and their findings that suggest V&L algorithms are relying on superficial correlations that enable them to achieve high performance in common situations but make them vulnerable when tested under different, but not especially unusual, conditions.

##### 3.3.1.1. VQA

Image-blind algorithms that only see questions often perform surprisingly well (Kafle and Kanan, 2016; Yang et al., 2016), sometimes even surpassing the algorithms having access to both (Kafle and Kanan, 2016). Algorithms also often provide inconsistent answers due to irrelevant changes in phrasing (Kafle and Kanan, 2017b; Ray et al., 2018), signifying a lack of question comprehension. When a VQA dataset is divided into different question-types, algorithms performed well only on easier tasks that CNNs alone excel at, e.g., detecting whether an object is present, but they performed poorly for complex questions that require bi-modal reasoning (Kafle and Kanan, 2017a). This discrepancy in accuracy is not clearly conveyed when simpler accuracy metrics are used. In a multi-faceted study, Agrawal et al. (2016) showed several quirks of VQA, including how VQA algorithms converge to an answer without even processing one half of the question and show an inclination to fixate on the same answer when the same question is repeated for a different image. Similarly, Goyal et al. (2017) showed that VQA algorithm performance deteriorates when tested on pairs of images that have opposite answers. As shown in Figure 2, VQA systems can actually amplify bias.

##### 3.3.1.2. Image captioning

In image captioning, simply predicting the caption of the training image with the most similar visual features yields relatively high scores using automatic evaluation metrics (Devlin et al., 2015). Captioning algorithms exploit multi-modal distributional similarity (Madhyastha et al., 2018), and generate captions similar to images in the training set, rather than learning concrete representations of objects and their properties.

##### 3.3.1.3. Embodied QA and visual dialog

EmbodiedQA ostensibly requires navigation, visual information collection, and reasoning, but Anand et al. (2018) showed that vision blind algorithms perform competitively. Similarly, visual dialog *should* require understanding both visual content and dialog history (Massiceti et al., 2018), but an extremely simple method produces near state-of-the-art performance for visual dialog, despite ignoring both visual and dialog information (Massiceti et al., 2018).

##### 3.3.1.4. Scene graph parsing

Predicting scene graphs requires understanding object properties and their relationships to each other. However, Zellers et al. (2018) showed that objects alone are highly indicative of their relationship labels. They further demonstrated that for a given object pair, simply guessing the most common relation for those objects in the training set yields improved results compared to state-of-the-art methods.

##### 3.3.1.5. RER

In a multi-faceted study of RER, Cirik et al. (2018) demonstrated multiple alarming issues. The first set of experiments involved tampering with the input referring expression to examine if algorithms properly used the text information. Tampering should reduce performance if algorithms make proper use of text to predict the correct answers. However, their results were relatively unaffected when the words were shuffled and nouns/adjectives were removed from the referring expressions. This signifies that it is possible for algorithms to get high scores without explicitly learning to model the objects, attributes and their relationships. The second set of experiments demonstrated that it is possible to predict correct candidate boxes for over 86% of referring expressions, without ever feeding the referring expression to the system. This demonstrates that algorithms can exploit regularities and biases in these datasets to achieve good performance, making these datasets a poor test of the RER task.

Some recent works have attempted to create more challenging datasets that probe the abilities to properly ground vision and language beyond shallow correlations. In FOIL (Shekhar et al., 2017), a single noun from a caption is replaced with another, making the caption invalid. Here the algorithm, must determine if the caption has been *FOILed* and then detect the *FOIL* word and replace it with a correct word. Similarly, in NLVR (Suhr et al., 2017), an algorithm is tasked with finding whether a description applies to a pair of images. Both tasks are extremely difficult for modern V&L algorithms with the best performing system on NLVR limited to around 55% (random guess is 50%), well short of the human performance of over 95%. These benchmarks may provide a challenging test bed that can spur the development of next-generation V&L algorithms. However, they remain limited in scope, with FOIL being restricted to noun replacement for a small number of categories (<100 categories from the COCO dataset). Hence, it does not test understanding of attributes or relationships between objects. Similarly, NLVR is difficult, but it lacks additional annotations to aid in the measurement of *why* a model fails, or eventually, why it succeeds.

#### 3.3.2. Lack of Interpretability and Confidence

Human beings can provide explanations, point to evidence, and convey confidence in their predictions. They also have an ability to say “I do not know” when the information provided is insufficient. However, almost none of the existing V&L algorithms are equipped with these abilities, making the models highly uninterpretable and unreliable.

In VQA, algorithms provide high-confidence answers even when the question is nonsensical for a given image, e.g., “What color is the horse?” for an image that does not contain a horse can yield “brown” with a very high confidence. Very limited work has been done in V&L to assess a system's ability to deal with lack of information. While Kafle and Kanan (2017a) proposed a class of questions called “absurd” questions to test a system's ability to determine if a question was unanswerable, they were limited in scope to simple detection questions. More complex forms of absurdity are yet to be tested.

Because VQA and captioning do not explicitly require or test for proper grounding or pointing to evidence, the predictions made by these algorithms remain uninterpretable. A commonly practiced remedy is to include visualization of attention maps for attention-based methods, or use post-prediction visualization methods such as Grad-CAM (Selvaraju et al., 2017). However, these visualizations shed little light on whether the models have “attended” to the right image regions. First, most V&L datasets do not contain attention maps that can be compared to the predicted attention maps; therefore, it is difficult to gauge the prediction quality. Second, even if such data were available, it is not clear what image regions the model *should* be looking at. Even for well-defined tasks such as VQA, answers to questions like “Is it sunny?” can be inferred using multiple image regions. Indeed, inclusion of attention maps does not make a model more predictable for human observers (Chandrasekaran et al., 2018), and the attention-based models and humans do not *look* at same image regions (Das et al., 2016). This suggests attention maps are an unreliable means of conveying interpretable predictions.

Several works propose the use of textual explanations to improve interpretability (Hendricks et al., 2016; Li et al., 2018). Li et al. (2018) collected text explanations in conjunction with standard VQA pairs and a model must predict both the correct answer and the explanation. However, learning to predict explanations can suffer from many of the same problems faced by image captioning: evaluation is difficult and there can be multiple valid explanations. Currently, there is no reliable evidence that such explanations actually make the model more interpretable, but there is some evidence of the contrary (Chandrasekaran et al., 2018).

Modular and compositional approaches attempt to reveal greater insight by incorporating interpretability directly into the design of the network (Hu et al., 2017; Johnson et al., 2017b, 2018). However, these algorithms are primarily tested on simpler, synthetically constructed datasets that lack the diversity of natural images and language. The exceptions that are tested on natural images rely on hand-crafted semantic parsers to pre-process the questions (Hu et al., 2017), which often over-simplify the complexity of the questions (Kafle and Kanan, 2017b).

#### 3.3.3. Lack of Compositional Concept Learning

It is hard to verify that a model has understood concepts. One method to do this is to use it in a novel setting or in a previously unseen combination. For example, most humans would not have a problem recognizing a purple colored dog, even if they have never seen one before, given that they are familiar with the concepts of purple and dog. Measuring such compositional reasoning could be crucial in determining whether a V&L system is a “horse.” This idea has received little attention, with few works devoted to it (Agrawal et al., 2017; Johnson et al., 2017a). Ideally, an algorithm should not show any decline in performance for novel concept combinations. However, even for CLEVR, which is composed of basic geometric shapes and colors, most algorithms show a large drop in performance for novel shape-color combinations (Johnson et al., 2017a). For natural images, the drop in performance is even higher (Agrawal et al., 2017).

## 4. Addressing Shortcomings

In this survey, we complied a wide range of shortcomings and challenges faced by modern V&L research based on the datasets and evaluation of tasks.

One of the major issues stems from the difficulty in evaluating if an algorithm is actually solving the task, which is confounded by hidden perverse incentives in modern datasets that cause algorithms to exploit unwanted correlations. Lamentably, most proposed tasks do not have built-in safeguards against this or even an ability to measure it. Many *post-hoc* studies have shed light on this problem. However, they are often limited in scope, require collecting additional data (Shekhar et al., 2017), or the modification of “standard” datasets (Kafle and Kanan, 2016; Agrawal et al., 2017, 2018). We outline prospects for future research in V&L, with an emphasis on discussing the characteristics of future V&L tasks and evaluation suites that are better aligned with the goals of a visual Turing test. Table 1 presents a short summary of challenges and potential solutions in V&L research.

**Table 1:** A summary of challenges and potential solutions for V&L problems.

**Shortcomings/challenges**	**Potential solutions**
Evaluation metrics are a poor measure for competence of algorithms due to dataset bias.	• Use metrics that account for dataset biases. • Carefully measure and report performance on individual abilities.
It is hard to tell if algorithms are “right for the right reasons.” They can perform well on benchmarks without actually solving the problem.	• Test the algorithms by withholding varying degrees of task-critical information from them to measure if they understand concepts. • Measure task understanding by asking the model to do the same task in dissimilar contexts and with alternative phrasing. • Develop defense mechanisms against “accidentally” reaching the correct solutions.
Trained systems are fragile and easily break when humans use them.	• Incorporate prediction confidence into evaluation. • Allow systems to output “I dont know.”
V&L Systems are one-trick-ponies, rarely able to generalize to more than one task.	• Create a V&L decathlon that tests numerous V&L tasks. Assess positive transfer among tasks.

### 4.1. New V&L Tasks That Measure Core Abilities

Existing V&L evaluation schemes for natural datasets ignore bias, making it possible for algorithms to excel on standard benchmarks without demonstrating proper understanding of underlying visual, linguistic or reasoning challenges. We argue that a carefully designed suite of tasks could be used to address this obstacle. We propose some possible approaches to improve evaluation by tightly controlling the evaluation of core abilities and ensuring that evaluation compensates for bias.

**Figure 4:**
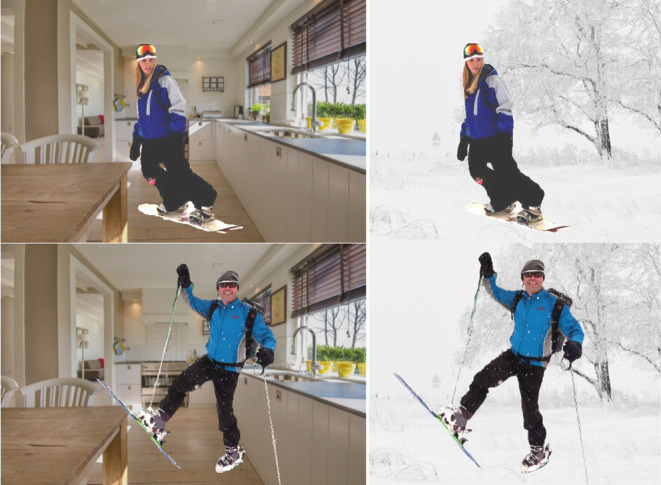
*Posters* dataset can help test bias. In this example, both contextual and gender bias are tested by placing out-of-context poster-cut-outs. Snowboarding is generally correlated with gender “male” and context “snow” (Hendricks et al., 2018).

CLEVR (Johnson et al., 2017a) enables measurement of compositional reasoning, but the questions and scenes have limited complexity. We argue that a CLEVR-like dataset for natural images could be created by composing scenes of natural objects (see Figure 4). This could be used to test higher-levels of visual knowledge, which is not possible in synthetic environments. This approach could be used to examine reasoning and bias-resistance by placing objects in unknown combinations and then asking questions with long reasoning chains, novel concept compositions, and distinct train/test distributions.Current benchmarks cannot reliably ascertain whether an algorithm has learned to represent objects and their attributes properly, and it is often easy to produce a correct response by “guessing” prominent objects in the scene (Cirik et al., 2018). To examine whether an algorithm demonstrates concept understanding, we envision a dataset containing simple queries, where given a set of objects and/or attributes as queries, the algorithm needs to highlight *all* objects that satisfy *all* of the conditions in the set, e.g., for *query={red}*, the algorithm must detect all red objects, and for *{red,car}*, it must detect all red cars. However, all queries would have *distractors* in the scene, e.g., {*red, car*} is only used when the scene also contains (1) cars that are non-red, (2) objects other than cars, or (3) other non-red objects. By abandoning the complexity of natural language, this dataset allows for the creation of queries that are hard to “guess” without learning proper object and attribute representations. Since the chance of a random guess being successful is inversely proportional to the number of *distractors*, the scoring can also be made proportional to *additional* information over a random guess. While this dataset greatly simplifies the language requirement, it would provide better measurement of elementary language grounded visual concept learning.

Similarly, the core abilities needed for language understanding can be tested using linguistic variations applied to the same visual input. Keeping the visual input unchanged can allow natural language semantic understanding to be better studied. Recent works have done this by rephrasing queries (Shah et al., 2019). To some extent, this can be done automatically by merging/negating existing queries, replacing words with synonyms, and introducing distractors.

We hope that carefully designed test suites that measure core abilities of V&L systems in a controlled manner will be developed. This serves as a necessary adjunct to more open-ended benchmarks, and would help dispel the “horse” in V&L.

### 4.2. Better Evaluation of V&L Systems

V&L needs better evaluation metrics for standard benchmarks. Here, we will outline some of the key points future evaluation metrics should account for:

- Evaluation should test individual skills to account for dataset biases (Kafle and Kanan, 2017a) and measure performance relative to “shallow” guessing (Kafle and Kanan, 2017b; Agrawal et al., 2018; Cirik et al., 2018).
- Evaluation should include built-in tests for “bad” or “absurd” queries (Kafle and Kanan, 2017a; Cirik et al., 2018).
- Test sets should contain a large number of compositionally novel instances that can be inferred from training but not directly matched to a training instance (Devlin et al., 2015; Johnson et al., 2017a).
- Evaluation should keep the “triviality” of the task in mind when assigning score to a task, e.g., if there is only a single cat then ‘Is there a black cat sitting between the sofa and the table?’ reduces to “Is there a cat?” for that image (Agrawal et al., 2016; Cirik et al., 2018).
- Robustness to semantically identical queries must be assessed.
- Evaluation should be done on questions with unambiguous answers; if humans strongly disagree, it is likely not a good question for a visual Turing test.

We believe future evaluation should probe algorithms from multiple angles such that a single score is derived from a suite of sub-scores that test different capabilities. The score could be divided into underlying core abilities (e.g., counting, object detection, fine-grained recognition, etc.), and also higher-level functions (e.g., consistency, predictability, compositionality, resistance to bias, etc).

### 4.3. V&L Decathlon

Most of the V&L tasks seek to measure language grounded visual understanding. Therefore, it is not unreasonable to expect an algorithm designed for one benchmark to readily transfer to other V&L tasks with only minor modifications. However, most algorithms are tested on single task (Kafle and Kanan, 2016; Yang et al., 2016; Yu et al., 2018), with very few exceptions (Anderson et al., 2018; Kim et al., 2018; Shrestha et al., 2019). Even within the same task, algorithms are almost never evaluated on multiple datasets to assess different skills, which makes it difficult to study the true capabilities of the algorithms.

To measure holistic progress in V&L research, we believe it is imperative to create a large-scale V&L decathlon benchmark. Work in a similar spirit has recently been proposed as DecaNLP (McCann et al., 2018), where many constituent NLP tasks are represented in a single benchmark. In DecaNLP, all constituent tasks are represented as question-answering for an easier input-output mapping. To be effective, a V&L decathlon benchmark should not only contain different sub-tasks and diagnostic information but also entirely different input-output paradigms. We envision models developed for a V&L decathlon to have a central V&L core and multiple input-output nodes that the model selects based on the input. Both training and test splits of the decathlon should consist of many different input-output mappings representing distinct V&L tasks. For example, the same image could have a **VQA question** “What color is the cat?,” a **pointing question** “What is the color of ‘that’ object?,” where “that” is a bounding box pointing to an object, and a **RER** “Show me the red cat.” Integration of different tasks encourages development of more capable V&L models. Finally, the test set should contain unanswerable queries (Kafle and Kanan, 2017a; Cirik et al., 2018), compositionally novel instances (Agrawal et al., 2017; Johnson et al., 2017b), pairs of instances with subtle differences (Goyal et al., 2017), equivalent queries with same ground truth but different phrasings, and many other quirks that allow us to peer deeper into the reliability and true capacity of the models. These instances can then be used to produce a suite of metrics as discussed earlier.

## 5. Conclusion

While V&L work initially seemed incredibly difficult, rapid progress on benchmarks made it appear as if systems would soon rival humans. In this article, we argued that much of this progress may be misleading due to dataset bias, superficial correlations and flaws in standard evaluation metrics. While this should serve as a cautionary tale for future research in other areas, V&L research does have a bright future. The vast majority of current V&L research is on creating new algorithms, however, we argue that constructing good datasets and evaluation techniques is just as critical, if not more so, for progress to continue. To this end, we outlined several potential solutions. First, we proposed the creation of diagnostic datasets that explicitly and carefully control for multiple sources of bias in vision and/or language. Next, we proposed the development of a large-scale benchmark consisting of a suite of V&L tasks that enable evaluation of various capabilities of algorithms on rich real-world imagery and natural language. V&L has the potential to be a visual Turing test for assessing progress in AI, and we believe that future research along the directions that we proposed will foster the creation of V&L systems that are trustworthy and robust.

## Author Contributions

CK and KK conceived of the scope of this review. KK, RS, and CK contributed to the text of this manuscript.

### Conflict of Interest

CK was employed by commercial company Paige. The remaining authors declare that the research was conducted in the absence of any commercial or financial relationships that could be construed as a potential conflict of interest.
